# Readthrough Approach Using NV Translational Readthrough-Inducing Drugs (TRIDs): A Study of the Possible Off-Target Effects on Natural Termination Codons (NTCs) on TP53 and Housekeeping Gene Expression

**DOI:** 10.3390/ijms242015084

**Published:** 2023-10-11

**Authors:** Riccardo Perriera, Emanuele Vitale, Ivana Pibiri, Pietro Salvatore Carollo, Davide Ricci, Federica Corrao, Ignazio Fiduccia, Raffaella Melfi, Maria Grazia Zizzo, Marco Tutone, Andrea Pace, Laura Lentini

**Affiliations:** Dipartimento di Scienze e Tecnologie Biologiche, Chimiche e Farmaceutiche (STEBICEF), Università degli Studi di Palermo, Viale delle Scienze Ed. 16-17, 90128 Palermo, Italy; riccardo.perriera@unipa.it (R.P.); emanuele.vitale@unipa.it (E.V.); piersalvo21@gmail.com (P.S.C.); davide.ricci@unipa.it (D.R.); federica.corrao01@unipa.it (F.C.); ignazio.fiduccia@unipa.it (I.F.); raffaella.melfi@unipa.it (R.M.); mariagrazia.zizzo@unipa.it (M.G.Z.); marco.tutone@unipa.it (M.T.); andrea.pace@unipa.it (A.P.)

**Keywords:** translational readthrough, nonsense, premature termination codons oxadiazole, precision medicine, genetic diseases

## Abstract

Nonsense mutations cause several genetic diseases such as cystic fibrosis, Duchenne muscular dystrophy, β-thalassemia, and Shwachman–Diamond syndrome. These mutations induce the formation of a premature termination codon (PTC) inside the mRNA sequence, resulting in the synthesis of truncated polypeptides. Nonsense suppression therapy mediated by translational readthrough-inducing drugs (TRIDs) is a promising approach to correct these genetic defects. TRIDs generate a ribosome miscoding of the PTC named “translational readthrough” and restore the synthesis of full-length and potentially functional proteins. The new oxadiazole-core TRIDs NV848, NV914, and NV930 (NV) showed translational readthrough activity in nonsense-related in vitro systems. In this work, the possible off-target effect of NV molecules on natural termination codons (NTCs) was investigated. Two different in vitro approaches were used to assess if the NV molecule treatment induces NTC readthrough: (1) a study of the translational-induced p53 molecular weight and functionality; (2) the evaluation of two housekeeping proteins’ (Cys-C and β2M) molecular weights. Our results showed that the treatment with NV848, NV914, or NV930 did not induce any translation alterations in both experimental systems. The data suggested that NV molecules have a specific action for the PTCs and an undetectable effect on the NTCs.

## 1. Introduction

During the translation of the messenger RNA (mRNA), the ribosome must stop at the natural stop codon (NTC) that is identified as the first stop codon found on the mRNA sequence. Stop codons are the nonsense UAG, UGA, and UAA nucleotide triplets, also known as *amber*, *opal*, and *ochre* codons, respectively [1].

When the ribosome reaches an NTC, the translation is stopped and the translation termination complex induces the release of nascent polypeptide. The translation termination complex is composed of two protein factors named eukaryotic release factors 1 and 3 (eRF1 and eRF3). The eRF1 factor mimics a tRNA and it enters the A site of the ribosome to bind and recognize the stop codon (all three stop codons). The eRF3 protein is a GTPase that, stimulated via the interaction with polyA-binding protein (PABP), modifies the conformation of eRF1, promoting the release of nascent polypeptide and the termination of the translation [2,3].

The coding mRNA sequence and the NTC are followed by an untranslated region, named 3′UTR, containing regulatory elements of the translation such as poly-A tail and multiple binding sites for specific regulatory proteins [4,5]. In addition, several in-frame nonsense codons can be found in the 3′UTR region of expressed genes [4].

Nonsense mutations result in the presence of an extra stop codon in the mRNA sequence upstream of the NTC. This anomalous stop codon inside the coding mRNA sequence is named the premature termination codon (PTC). The PTCs are recognized as canonical stop signals by the translation termination complex and that induces an early termination of the translation. The presence of the PTC is thus involved in the synthesis of truncated and dysfunctional proteins that are degraded by cellular pathways [6].

The deleterious effects of the presence of the PTCs are in part recovered by the nonsense-mediated decay pathway (NMD) degrading mRNAs that contain premature stop codons to prevent the production of altered proteins [7].

Moreover, it was observed that, in physiological conditions, a near-cognate tRNA can be inserted in the A site of the ribosome during the translation process via non-programmed translational readthrough. The percentage of non-programmed translational readthrough probability is less than 0.1% and depends on the identity of the stop codon [1,8,9].

Stop codon readthrough efficiency can be influenced by different factors such as stop codon identity, the nucleotides surrounding it (genetic context), protein isoforms, and specific mRNAs [5,9]. For example, the first nucleotide immediately following the stop codon strongly influences the termination efficiency. A purine in this position increases the translation termination in 90% of the most expressed genes; indeed, a pyrimidine near-stop codon facilitates translational readthrough [9,10]. Anyway, the amount of the protein synthesized following a non-programmed readthrough event is really small compared to the product of the physiological translation termination process.

Several genetic diseases are caused by nonsense mutations. Among the most diffused nonsense-related diseases there are cystic fibrosis, Duchenne muscular dystrophy, choroideremia, β-thalassemia, primary immune deficiencies, Shwachman–Diamond syndrome, and some inherited cancer typologies (e.g., breast cancer, ovarian cancer, adenocarcinoma) [11,12,13,14,15,16,17,18,19,20].

A strategy to restore the correct translation of the proteins deriving from the expression of nonsense-mutated genes is nonsense suppression therapy using translational readthrough-inducing drugs (TRIDs). Different classes of compounds such as geneticin (also known as G418), ELX-02, gentamicin, paromomycin, neomycin, diaminopurine (DAP), and PTC124 (Ataluren) have the ability to induce ribosome translational readthrough [21,22,23]. This mechanism consists of the misreading of the PTC during translation by TRIDs and in the insertion of a near-cognate tRNA instead of the eRF1 factor. This results in the synthesis of a full-length protein and in the rescue of its function [10].

Only the readthrough mechanism of action of the aminoglycoside antibiotics, such as G418, is well clarified. These classes of compounds interact with the minor subunit of the eukaryotic ribosome inside the decoding center, increasing the miscoding errors at the PTCs and the recruitment of near-cognate tRNA [24].

Recently, some evidence about the Ataluren (PTC124) mechanism of action was described. According to Huang S. and colleagues, Ataluren could bind two different sites during translation: the 18S rRNA near the decoding center and the PTC in the mRNA [25,26].

**Scheme 1:**
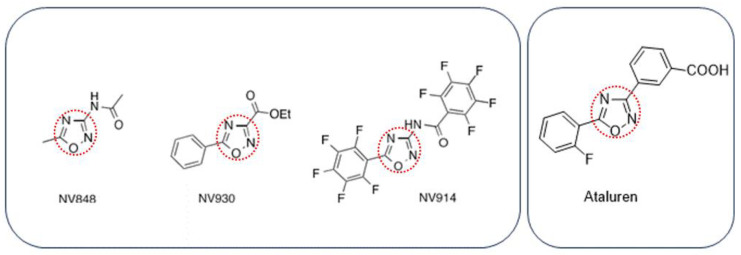
NV molecules chemical structures, the red circle indicates the common oxadiazole-core.

In the last few years, three new TRIDs were identified by our group and tested to validate their translational readthrough ability and tolerability in vivo [27,28,29]. These compounds, named NV848, NV914, and NV930 (also known as NV molecules; PTC Int. Appl. WO 2019/101709 A1 20190531), present an oxadiazole-core chemical structure, like the Ataluren core, but with different functional groups (Scheme 1).

NV molecules showed a good translational readthrough activity in cystic fibrosis in in vitro systems and a suitable tolerability in in vivo experiments [27,28]. Recently, we explored the possible MOA of these molecules to identify the interaction with specific proteins involved in the translation process. Our results showed that NV914 and NV930 could interact with the FTSJ1 2′-O-methyltransferase [29].

An important aspect of the functionality and efficiency of the TRIDs is their specific action against PTCs. The possible readthrough action and suppression of the NTCs by TRIDs is considered an off-target that hypothetically alters the translational process of the cell.

The purpose of this work is to investigate the possible NV848, NV914, and NV930 readthrough effects on NTCs, and to validate the specific action of these compounds on PTCs. Two different in vitro approaches were used to assess the translation alteration of different proteins after treatment with NV molecules.

The first one was the study of the molecular weight and functionality of the p53 protein, used as an inducible protein model. Its translational increase was stimulated via DNA damage response induction in combination with the treatment with NV848, NV914, or NV930. The full-length p53 mutants are known to be thermodynamically unstable; therefore, the loss of activity of the transcription factor can be considered an indicator of the uncorrected translation of the protein [30].

The second approach was the evaluation of two housekeeping proteins’ (Cys-C and β2M) molecular weights after treatment with NV molecules. The evaluation of translational errors using these housekeeping proteins was previously used in a study concerning the ELX-02 readthrough effect [31].

## 2. Results

### 2.1. Evaluation of p53 Molecular Weight Alteration after DNA Damage Induction Mediated by Doxorubicin Combined with NV848, NV914, or NV930 Treatment

To exclude the possible off-target effects on NTCs by NV848, NV914, or NV930, p53 was chosen as a model of an inducible protein to analyze its molecular weight after the induction of DNA damage in association with NV molecule treatment. In fact, the eventual NTC readthrough could cause the production of longer proteins with a molecular weight higher than that of wild-type p53.

p53 is one of the best-characterized proteins in the research field since it is involved in cell cycle arrest, DNA repair, senescence, and apoptosis, all processes involved in cancer [32]. In order to exert its proper function, this protein has to undergo a series of important events: aggregation in tetramer form, phosphorylation of specific amino acidic residues, nuclear localization, and interaction with transcription-promoting DNA sequences of specific genes [33].

One of the earliest genes activated by the p53 transcriptional factor in response to DNA damage is *CDKN1A* [34]. This gene encodes for a cyclin-dependent kinase inhibitor, also known as p21, that is fundamental to arresting the cell cycle during the DNA repair response [35].

Immediately 24 h after DNA damage, p53 mRNAs and proteins are stabilized and the translation of p53 is increased in order to accelerate the DNA damage response [36]. p53 alterations can interfere with the functionality and correct localization of this protein in several ways [37].

Based on these observations, to evaluate a possible readthrough activity of the NV molecules (NV848, NV914, and NV30) on the NTCs during the translation of the p53 protein, DNA damage was induced via Doxorubicin in HCT116 cells, in the presence of NV molecules. The purpose of these experiments was to visualize the higher molecular weight forms of p53 eventually expressed after treatment with NV molecules for 24 h. Moreover, the experiment aimed to assess the presence of the not-functional forms of the p53 protein due to an incorrect translational process.

HTC116 cells are colon cancer-derived cells with a high replication rate. These cells were used because of their higher translation capacity compared to normal cells.

**Figure 1:**
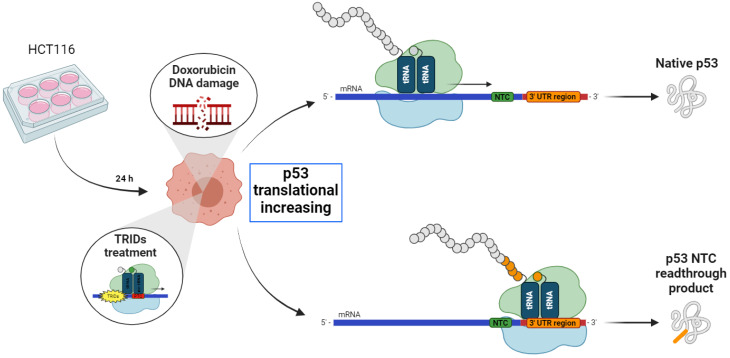
Experimental scheme. Doxorubicin DNA damage induces p53 expression in HCT116 cells. NV molecules could generate translational readthrough of the natural mRNA termination codons (NTCs), even on p53 NTC. This miscoding error could result in alterations in protein expression, nuclear localization, and/or DNA/protein interaction.

Twenty-four hours after plating, HCT116 cells were treated with NV molecules in combination with Doxorubicin, which induce DNA damage and p53 translation increase, as shown in the graphic representation in Figure 1.

p53 protein expression, functionality, and localization were analyzed after the DNA damage induction in the presence of NV848, NV914, or NV930.

**Figure 2:**
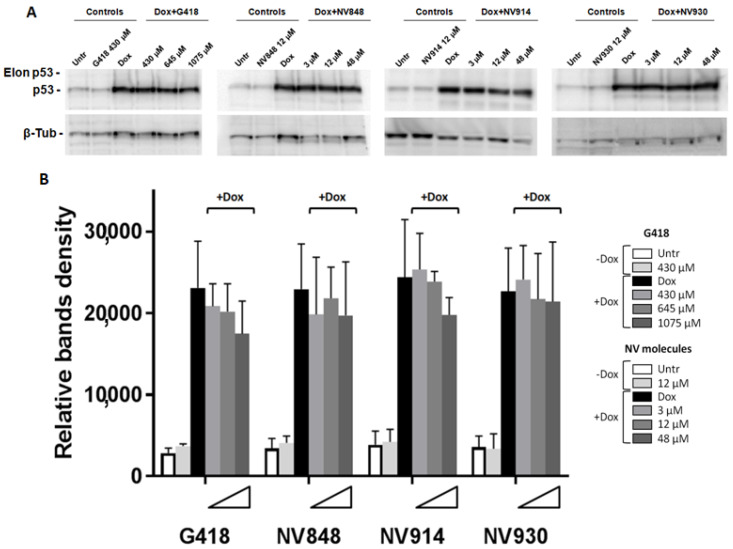
(**A**) Western blot analysis to detect p53 in HCT116 cells 24 h after treatment with G418 (430 μM, 645 μM, and 1,075 μM) or NV molecules (3 μM, 12 μM, and 48 μM) at increasing concentrations, in combination with Doxorubicin (Dox, 0.2 μg/mL). Only compound indicated the treatment without DNA damage induction (-Dox). NV or G418 samples indicated the treatment with different TRID concentrations (triangles) in combination with Doxorubicin (+Dox). Beta-tubulin (β-Tub) was used as a loading control. (**B**) Western blot band quantification was performed by ImageJ software (Rasband, W.S., ImageJ, U. S. National Institutes of Health, Bethesda, Maryland, USA, https://imagej.nih.gov/ij/, 1997–2018).

In particular, HCT116 cells were seeded in 6-well plates and treated with Doxorubicin 0.2 μg/mL in combination with G418 or NV molecules at the indicated concentrations (**G418:** 430 μM, 645 μM, and 1075 μM; **NV848**, **NV914**, and **NV930:** 3 μM, 12 μM, 48 μM; Figure 2).

The G418 aminoglycoside was used as a positive control considering its capacity for inducing NTC genome-wide readthrough [10].

Western blot experiments and bands quantification analysis showed a little decrease in the p53 protein levels in HCT116 treated with G418 and NV914 after DNA damage compared to the Doxorubicin (Dox) samples (black bars in Figure 2B). However, in all the analyzed samples, p53 bands with higher molecular weights (Elon p53) were not detectable.

### 2.2. Study of p53 Protein Localization and Functionality after DNA Damage Response and NV Molecule Treatment

In response to DNA damage, the p53 protein is stabilized and its expression is increased. Post-translational modifications and other signals are involved in p53 tetramerization and nuclear localization, which is needed to activate specific gene transcription.

Nuclear translocation is regulated by the presence of a protein nuclear localization sequence (NLS) that, together with export sequences and the tetramerization domain, is located in the C-terminal region of p53. The suggestion is that if any alteration in the p53 translation is induced by NV molecule treatment, protein functions would be impaired as well as nuclear localization.

**Figure 3:**
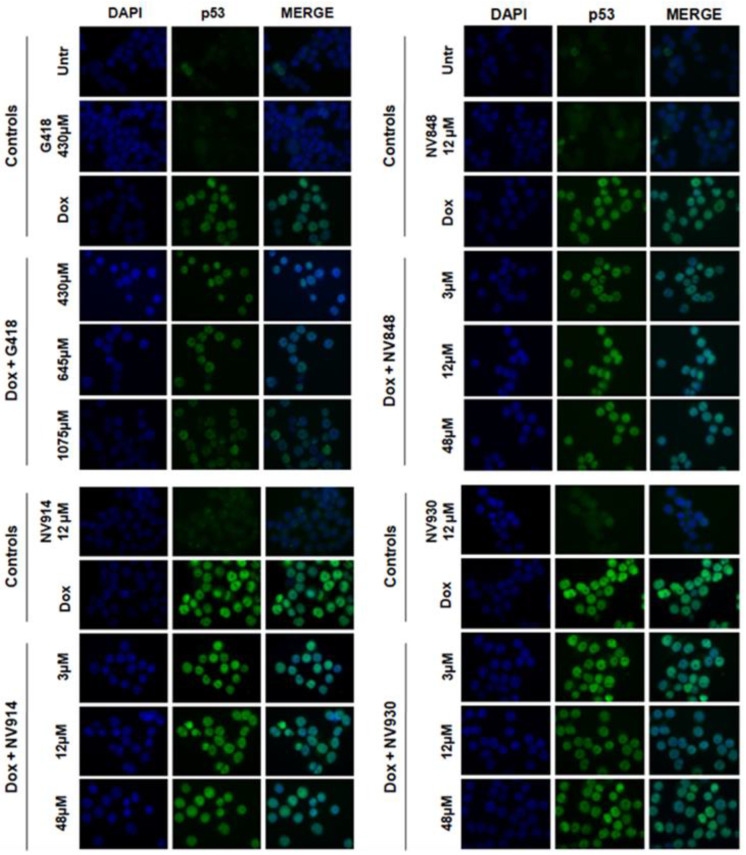
Immunofluorescence analysis of HCT116 to visualize the localization of p53 (green) 24 h after DNA damage induction by Doxorubicin (0.2 μg/mL) and after 24 h of treatment with G418, NV848, NV914, or NV930 at the indicated concentrations. p53 protein (green) was revealed by a specific primary antibody and a fluorochrome-conjugated secondary antibody (Alexa-488). Nuclei (blue) were stained with DAPI (4′,6-diamidino-2-phenylindole). G418 was used as a positive control of NTC readthrough.

**Figure 4:**
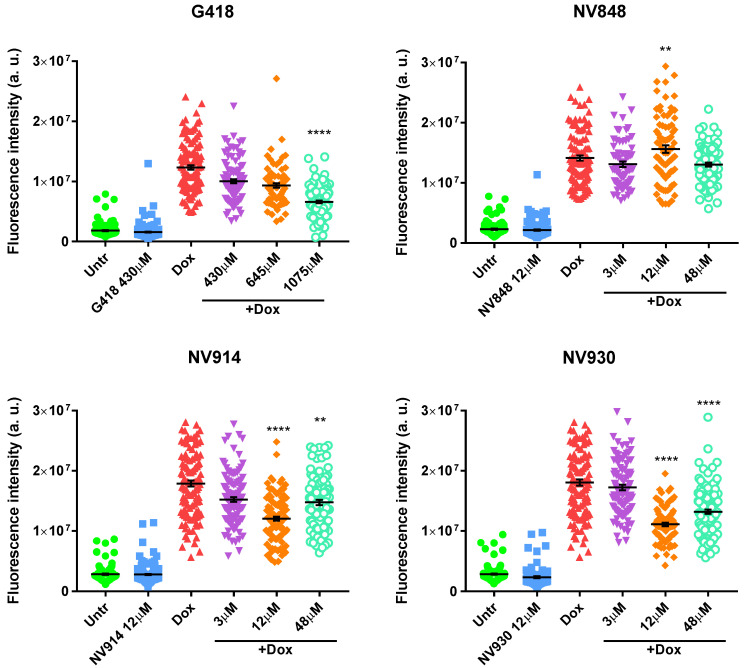
Quantification of the p53 signal relative to the immunofluorescence analysis. The single shapes indicate the amount of fluorescence of a single cell (arbitrary units). Fluorescence intensity quantification was performed by ImageJ software (Rasband, W.S., ImageJ, U. S. National Institutes of Health, Bethesda, Maryland, USA, https://imagej.nih.gov/ij/, 1997–2018). Samples were analyzed compared to DNA damage controls only treated with Doxorubicin (Dox). Data were analyzed by GraphPad Prism 7 software. A probability value (*p*): two symbols (**) for *p* < 0.01, four symbols (****) for *p* < 0.0001.

In order to visualize p53 protein localization after DNA damage induction and TRID treatments, the immunofluorescence assay was performed. HCT116 cells were treated with Doxorubicin and simultaneously with G418 or NV molecules at the indicated concentrations for 24 h (Figure 3 and Figure 4).

Interestingly, the samples treated with Doxorubicin and 1075 μM of G418 showed a decreased fluorescence intensity similar to the controls where DNA damage was not induced (Untr and G418 430 μM; Figure 4). This result could be indicative of the reduced ability of the p53 protein to respond against DNA damage as a consequence of aberrant protein production. Cells treated with NV848 and Doxorubicin showed a similar fluorescence intensity compared to the DNA-damaged positive control samples (Dox).

On the other hand, samples treated with NV914 and NV930 showed a decreased fluorescence signal at 12 μM. However, the p53 fluorescent signal was always localized in the cell nuclei in all samples treated with Doxorubicin and in combination with TRIDs (G418 and NV molecules).

Since no significant change in p53 nuclear localization was revealed after treatment with Doxorubicin and NV molecules, the transcription levels of p21 (*CDKN1A*) were analyzed in order to confirm the functionality of the p53 protein.

**Figure 5:**
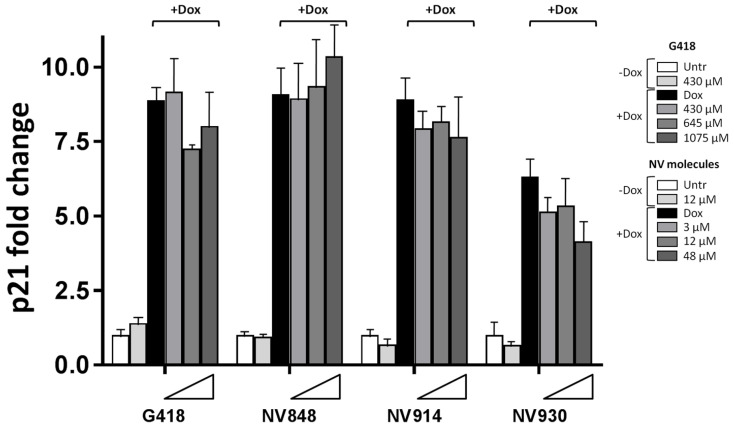
Real-time RT-PCR of the p21 (CDKN1A) mRNA in HCT116 cells, untreated (Untr) or treated, with G418 (430 μM, 645 μM, 1075 μM) or NV molecules (3 μM, 12 μM, 48 μM) after DNA damage induction by Doxorubicin (Dox, 0.2 μg/mL). Only compound indicated the treatment without DNA damage induction (-Dox). NV or G418 samples indicated the treatment with different TRID concentrations (triangles) in combination with Doxorubicin (+Dox). Analyses were performed in triplicate 24 h after treatment conditions (n = 2).

HCT116 cells were treated with Doxorubicin and NV molecules, and the total mRNA was extracted and analyzed via Real-Time RT-PCR to determine the p21 (*CDKN1A*) mRNA expression levels (Figure 5).

A sensible p21 mRNA reduction (around 16%) was observed in the presence of DNA damage and high concentrations of the aminoglycoside G418 at 645 μM (Figure 5).

### 2.3. Molecular Weight Analysis of the Two Housekeeping Proteins (Cystatin-C and β-2-Microglobulin) after Treatment with NV848, NV914, or NV930, to Detect the Possible NTC Readthrough Miscoding

Recently, a new molecule, named ELX-02, has been established to be effective in treating nonsense mutations and to be safe. Furthermore, it has been shown that its targets are specifically PTCs. In fact, to demonstrate all the above-mentioned characteristics of this TRID, Crawford et al. have developed a method similar to the one previously shown for the p53 protein [31].

Precisely, they considered two housekeeping proteins, Cystatin-C and β-2-Microglobulin, that have the molecular weight of 15 kDa and 13 kDa, respectively.

The rationale of the used method is the same as described for p53. Indeed, if TRID treatment induces the readthrough of NTCs in Cystatin-C and β-2 Microglobulin mRNAs, a higher molecular weight should be detected via Western blot analysis.

**Figure 6:**
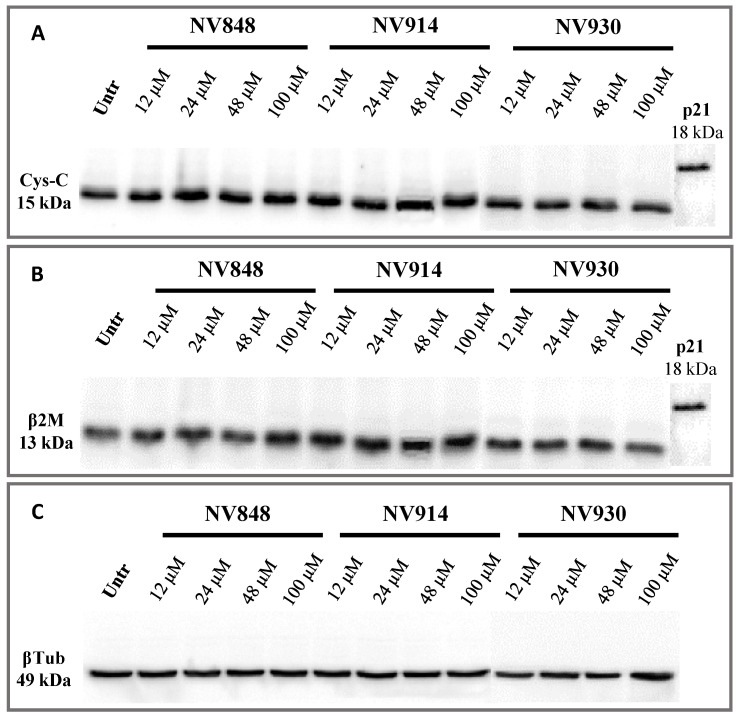
Western blot analysis in 16HBE cells untreated (Untr; negative control) or treated after 24 h with NV848, NV914, and NV930 at indicated concentrations. Images show the molecular weights of two housekeeping proteins, Cystatin-C (Cys-C; **A**) and β-2-microglobulin (β2M; **B**). p21 protein was used as an internal control of expected high molecular weight. β-tubulin (βTub) was included as a loading control (**C**). Images were derived from different membranes, and they were placed according to molecular weight marker migration.

On the basis of the experiment performed by Crawford et al., human bronchial epithelial (16HBE) cells were treated with increasing concentrations of NV848, NV914, and NV930 molecules (Figure 6) [31]. The choice of this new cell model system was made since it is frequently used in cystic fibrosis research.

**Figure 7:**
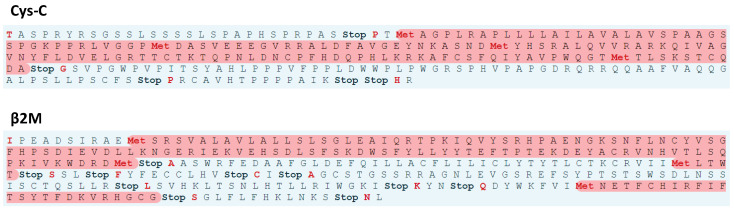
In silico translation of Cystatin-C (Cys-C) and β-2-microglobulin (β2M) mRNAs. Open riding frames are highlighted in red.

In particular, cells were treated for 24 h at 12, 24, 48, or 100 µM, then, proteins were extracted and analyzed via Western blot. The p21 protein detection was used as an internal control of the known molecular weight, similar to the hypothetical molecular weight of the two proteins, Cystatin-C and β-2 Microglobulin, if the natural stop codon was translated (Figure 7).

**Figure 8:**
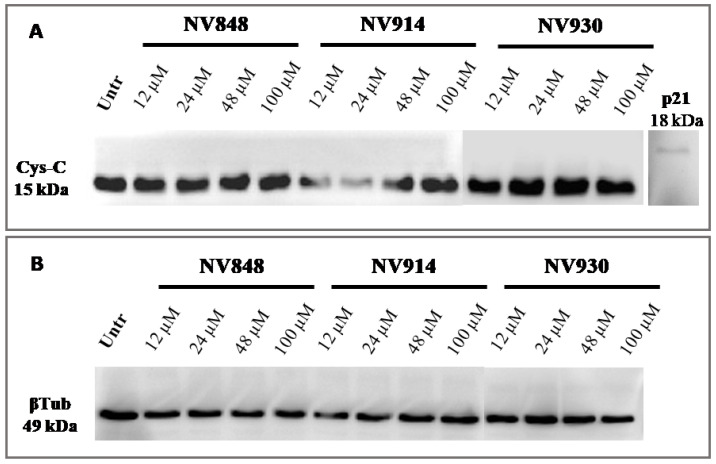
Western blot analysis in 16HBE cells untreated (Untr; negative control) or treated after 72 h with NV848, NV914, and NV930 at indicated concentrations. Every 24 h, the treatments were refreshed. Images show the molecular weights of cystatin-C (Cys-C; **A**). p21 protein was used as an internal control of expected high molecular weight. Β-tubulin (βTub) was included as a loading control (**B**). Images were derived from different membranes and they were placed according to molecular weight marker migration.

In addition, the same experiment was performed with prolonged (72 h) treatment with NV molecules to validate the absence of NTC readthrough (Figure 8).

No bands relative to the cystatin-C proteins with a higher or abnormal molecular weight are visible, confirming the data obtained after 24 h of treatment with NV molecules.

These experiments confirm that NV molecules do not induce a significant readthrough on natural stop codons.

## 3. Discussion

The three new TRIDs, NV848, NV914, and NV930, result in being good compounds in order to induce translational readthrough in cystic fibrosis cellular systems and are well tolerated in vivo [27,28]. According to the experimental evidence, NV molecules restore CFTR protein expression after the treatment of in vitro cystic fibrosis model systems and Shwachman–Diamond model systems [15,28]. Recently, we showed that two of our NV molecules, NV914 and NV930, could interact with the FTSJ1 2′-O-methyltransferase to favor the readthrough process [29].

Advanced studies about the main off-target effects of readthrough-inducing compounds are proposed in this work. The specificity of NV848, NV914, and NV930 against PTCs and the possibility of NTC readthrough are, respectively, fundamental and limiting factors for the TRID’s efficiency.

We used two different approaches to investigate the possible NV molecule NTC readthrough:

The evaluation of the p53 protein activity after DNA damage induction and the presence of elongated proteins produced by housekeeping genes (Cys-C and β2-microglobulin). The DNA damage induction by Doxorubicin causes a temporal-induced activation of the p53 protein translation [36,37]. According to the experimental hypothesis, if the combined treatment with Doxorubicin and NV848, NV914, or NV930 would have induced p53 NTC readthrough, we could expect p53 molecular weight alterations. However, the Western blot analyses do not show any p53 protein form with an increased molecular weight.

In order to exclude the possibility of p53 C-terminal functional domain alterations, the protein localization and transcriptional activation ability after DNA damage induction were investigated. No significant changes were detected relative to the p53 nuclear localization and *CDKN1A* (p21) activation.

Interestingly, the fluorescence intensity of the p53 protein after DNA damage induction and G418 treatment was comparable to the controls without doxorubicin treatment in some cells. These data could suggest that the G418 NTC readthrough partially altered the localization of p53 during the DNA damage response. We interpreted this result as being indicative of a possible off-target effect of the antibiotic, even if it is not trivial to evidence the effect due to the toxicity of the molecule at these concentrations. Relative to NV930, our experiments have shown a similar effect at 12 and 48 μM. The principal difference between the G418 treatment and NV930 is the absence of cell mortality in the presence of the NV930 molecule compared to the G418.

Molecular weight studies of the two housekeeping proteins, Cystatin-C and β-2-Microglobulin, are proven methodologies to analyze NTC readthrough by TRIDs [31].

In silico evaluations of the possible molecular weight increase for the Cystatin-C and β-2-Microglobulin revealed an increase of 7.32 kDa and 5.71 kDa, respectively, for the two proteins (ExPASy translate tool: https://web.expasy.org/translate/ (accessed on 3 November 2021); ExPASy compute pI/Mw tool: https://web.expasy.org/compute_pi/ (accessed on 3 November 2021)). We included, for these analyses, the sense codons present between the NTC of the Cystatin-C and β-2-Microglobulin and the next stop codon in the 3′UTR regions.

After 24 and 72 h of treatments, NV848, NV914, or NV930 in a normal cellular system (16HBE) did not induce any molecular weight increase in the two proteins.

These two presented methodologies, especially the studies conducted on p53 protein expression and functionality, are alternative investigations for the evaluation of protein alterations mediated by compounds with the possible ability to induce NTC readthrough. In these experiments, we analyzed the protein samples and their alterations in contrast to ribosome profiling in which the ribosome position is evaluated [10]. Moreover, the protein analyses in combination with ribosome profiling could give complementary information regarding the possible NTC readthrough mediated by TRIDs.

## 4. Materials and Methods

### 4.1. Compounds

Compounds were prepared and purchased, as reported in Table 1.

### 4.2. Cell Culture and Conditions

All cells were cultured in a humidified incubator with an atmosphere of 5% CO_2_ at 37 °C.

HCT116 (colon cancer cells) cells were cultured in DMEM (Dulbecco’s modified eagle medium, GIBCO, Waltham, MA, USA) supplemented with 10% FBS (fetal bovine serum, GIBCO) and 1% Streptomycin and Penicillin antibiotics (Corning). Antibiotics were removed 24 h before treatments.

Human bronchial epithelial cells (16HBE) were cultured in MEM (minimum essential medium, GIBCO, Waltham, MA, USA) supplemented with 10% FBS (fetal bovine serum, GIBCO) and 1% Streptomycin and Penicillin antibiotics (Corning, NY, USA). Before plating, every plate was coated with rat tail collagen (1:100). Antibiotics were removed 24 h before treatments.

### 4.3. Western Blotting

Protein samples were extracted from cellular pellets (10^6^ cells for each sample) using RIPA buffer (ThermoScientific, Waltham, MA, USA) and protease cocktail inhibitor (1:100, ThermoScientific, Waltham, MA, USA) at 4 °C. After extraction, proteins were quantified via the Bradford assay method (Coomassie blue dye, ThermoScientific, Waltham, MA, USA) and samples were compared to a BSA (bovine serum albumin) protein standard curve at known concentrations.

For the analysis of p53, Cystatin-C (Cys-C) and β-2-Microglobulin (β2M) proteins (20 μg) were separated in 12% SDS-PAGE gel and transferred to a PVDF transfer membrane overnight at 4 °C and 12 V (constant voltage). Blotted membranes were blocked with non-fat dry milk 5% (1 h at room temperature) and after that, membranes were incubated with primary antibody anti-p53 (mouse, p53 DO-1, Santa Cruz, CA, USA, 1:2000), primary antibody anti-Cystatin-C (rabbit, Cell Signaling Biotechnology, Danvers, MA, USA B1:1000), or primary antibody anti-β-2-Microglobulin (rabbit, Cell Signaling Biotechnology, Danvers, MA, USA, 1:1000) overnight at 4°C. Anti-β Tubulin primary antibody (mouse, Sigma Aldrich, St. Louis, MO, USA 1:5000) was used to detect β-tubulin (βTub) as a loading control to normalize the protein bands.

After three washes (15 min on shaker) with TBS-Tween^TM^-20 1X (ThermoScientific, Waltham, MA, USA), Waltham CA, USA), membranes were incubated with anti-mouse (ThermoScientific, Waltham, MA, USA), 1:5000) or anti-rabbit (Promega, Madison, WI, USA 1:2500) HRP-conjugated secondary antibodies for 1 h. After incubation, the membranes were washed three times (15 min on a shaker) with TBS-Tween^TM^-20 1X (ThermoScientific, Waltham, MA, USA). The detection of the bands was performed by SuperSignal^®^ West Femto kit (ThermoScientific, Waltham, MA, USA) and images were acquired by ChemiDoc MP imaging system (Bio-Rad, Hercules, CA, USA). Gel bands were quantified by ImageJ software (Rasband, W.S., ImageJ, U. S. National Institutes of Health, Bethesda, Maryland, USA, https://imagej.nih.gov/ij/, 1997–2018).

### 4.4. Immunofluorescence Microscopy

In total, 5 × 10^4^ HCT116 cells were grown on round glass coverslips in 12-well plates with 1 mL of medium (without antibiotics). After removing the medium and one wash in DPBS 1X (Dulbecco’s phosphate buffer saline, GIBCO, Waltham, MA, USA), cells were fixed with cold methanol for 1 min and treated with Triton-X 0.01% for 10 min at room temperature. After washing, fixed cells were blocked in BSA 0.1% for 1 h and incubated with primary antibody anti-p53 (mouse, p53 DO-1, Santa Cruz Biotechnology, CA, USA, 1:2000) overnight at 4 °C. Coverslips were then incubated with a goat polyclonal to mouse Alexa Fluor-488 (Abcam, Cambridge, GB 1:1000) secondary antibody for 1 h at room temperature.

Nuclei were stained with ProLong^TM^ Gold antifade mounting medium with DAPI (ThermoScientific, Waltham, MA, USA). Cells were observed using a 63X objective in a Zeiss Axioskop microscope equipped for fluorescence. Fluorescence signals were quantified by ImageJ software (Rasband, W.S., ImageJ, U. S. National Institutes of Health, Bethesda, Maryland, USA, https://imagej.nih.gov/ij/, 1997–2018).

### 4.5. Real-Time RT-PCR

Total RNA was extracted from the cellular pellet (3 × 10^5^ cells for sample) by using the RNeasy^®^ Mini Kit (QIAGEN, Hilden, DE) according to the manufacturer’s instructions and samples were purified by DNase Max^®^ kit (QIAGEN, Hilden, DE). RNA was reverse transcribed in a final volume of 50 μL using the High-Capacity cDNA Reverse Transcription Kit (Applied Biosystems, Waltham, MA, USA). For each sample, 2 μL of cDNA, corresponding to 100 ng of reverse-transcribed RNA, was analyzed via real-time RT-PCR (95 °C for 15 s, 60 °C for 60 s, repeated for 40 cycles) in triplicate, using AB PRISM 7300 instrument (Applied Biosystems, Waltham, MA, USA). Real-time RT-PCR was performed in a final volume of 25 μL comprising 1X Master Mix SYBR Green (Applied Biosystems, Waltham, MA, USA) and 1 μM of forward and reverse primers, which were the following:

-p21 Fwd= 5′-CTG GAG ACT CTC AGG GTC GA-3′,
Rev: 5′-CGG ATT AGG GCT TCC TCT TG-3′;
-GAPDH Fwd= 5′-CTC ATG ACC ACA GTC CAT GCC-3′,
Rev= 5′-GCC ATC CAC AGT CTT CTG GGT-3′.

Data were analyzed using triplicate values of C_t_ (cycle threshold). Levels of RNA were determined by using the SDS software version (Applied Biosystems, Waltham, MA, USA) according to the 2^−ΔΔCt^ method and C_t_ values were normalized to the internal control GAPDH.

### 4.6. Statistics

All data are expressed as mean values ± standard error of the mean (SEM). Statistical analysis was performed by Student’s t-test and one-way ANOVA when appropriate by GraphPad Prism software (Inc., La Jolla, CA, USA) version 7.0.0 for Windows. A probability value (*p*) of less than 0.05 was regarded as significant and indicated in relevant graphs as one symbol (*) for *p* < 0.05, two symbols (**) for *p* < 0.01, three symbols (***) for *p* < 0.001, and four symbols (****) for *p* < 0.0001.

## 5. Conclusions

Our work represents an explorative study of the possible off-target effects of the NV848, NV914, and NV930 molecules. The treatment with the three molecules does not induce any evidential p53 molecular weight alterations after the combined treatment with Doxorubicin. Although we are aware of the limits of the used approach, the p53-mediated DNA damage response (p53 nuclear localization and *CDKN1A* expression) does not seem to be altered by the NV848 and NV914 molecule treatments, which is in contrast to a reduction in p53 functionality after G418 treatment, which is known to induce NTC translation [38], and, in part, for NV930.

The results of our molecular weight analyses of two housekeeping proteins (Cystatin-C and β-2-Microglobulin) after NV848, NV914, or NV930 treatments confirm the absence of elongated protein products in a normal cellular system as 16HBE cells.

In conclusion, we collected evidence about the possible off-target effects of NV848, NV914, and NV930, suggesting that these TRIDs do not have appreciable NTC readthrough effects in this cell model system, despite the readthrough effect previously observed for the PTCs. Our conclusions do not exclude a potential NTC readthrough effect for other mRNAs, but these findings were obtained in high translational rate proteins (housekeeping and translational induced proteins), increasing the possibility of an eventual interaction between NV molecules and these mRNAs. Our work is a first step in comprehending the off-target effects of our TRIDs and other experiments are necessary to deepen the knowledge of the identified molecules. The experimental design presented in this work or other similar approaches could be informative about NTC readthrough translation in future studies.

## 6. Patents

Pibiri, A. Pace, M. Tutone, L. Lentini, R. Melfi, A. Di Leonardo, Oxadiazole Derivatives for the Treatment of Genetic Diseases Due to Nonsense Mutations, PCT Int. Appl. (2019), WO 2019/101709 A1 20190531.

## Figures and Tables

**Table 1:** Compounds were dissolved in DMSO (dimethyl sulfoxide, Sigma Aldrich, St. Louis, MO, USA)) or sterile water (H_2_O) at the indicated stock concentrations. All compound stocks were stored at −20 °C. (*) TRIDs were produced at the STEBICEF Department, University of Palermo, Palermo, according to the procedure reported in Pibiri I., et al. 2020 [27].

Compound	Solvent	Concentration	Manufacturer
Geneticin (G418)	Water	200 mg/mL	Gibco
NV848	DMSO	100 mM	*
NV914	DMSO	100 mM	*
NV930	DMSO	100 mM	*

## Data Availability

The data presented in this study are openly available in: https://iris.unipa.it/.

## References

[1] Palma M., Lejeune F. (2021). Deciphering the molecular mechanism of stop codon readthrough. Biol. Rev..

[2] Beißel C., Neumann B., Uhse S., Hampe I., Karki P., Krebber H. (2019). Translation termination depends on the sequential ribosomal entry of eRF1 and eRF3. Nucleic Acids Res..

[3] Neu-Yilik G., Raimondeau E., Eliseev B., Yeramala L., Amthor B., Deniaud A., Huard K., Kerschgens K., Hentze M.W., Schaffitzel C. (2017). Dual function of UPF3B in early and late translation termination. EMBO J..

[4] Yamashita A., Takeuchi O. (2017). Translational control of mRNAs by 3′-Untranslated region binding proteins. BMB Rep..

[5] Wu C., Roy B., He F., Yan K., Jacobson A. (2020). Poly(A)-Binding Protein Regulates the Efficiency of Translation Termination. Cell Rep..

[6] Morais P., Adachi H., Yu Y.-T. (2020). Suppression of Nonsense Mutations by New Emerging Technologies. Int. J. Mol. Sci..

[7] Drummond D.A., Wilke C.O. (2009). The evolutionary consequences of erroneous protein synthesis. Nat. Rev. Genet..

[8] Dabrowski M., Bukowy-Bieryllo Z., Zietkiewicz E. (2015). Translational readthrough potential of natural termination codons in eucaryotes—The impact of RNA sequence. RNA Biol..

[9] Beryozkin A., Nagel-Wolfum K., Banin E., Sharon D. (2023). Factors Affecting Readthrough of Natural Versus Premature Termination Codons. Adv. Exp. Med. Biol..

[10] Wangen J.R., Green R. (2020). Stop codon context influences genome-wide stimulation of termination codon readthrough by aminoglycosides. eLife.

[11] Laselva O., Guerra L., Castellani S., Favia M., Di Gioia S., Conese M. (2022). Small-molecule drugs for cystic fibrosis: Where are we now?. Pulm. Pharmacol. Ther..

[12] Morkous S.S. (2020). Treatment with Ataluren for Duchene Muscular Dystrophy. Pediatr. Neurol. Briefs.

[13] Sanchez-Alcudia R., Garcia-Hoyos M., Lopez-Martinez M.A., Sanchez-Bolivar N., Zurita O., Gimenez A., Villaverde C., da Silva L.R.-J., Corton M., Perez-Carro R. (2016). A Comprehensive Analysis of Choroideremia: From Genetic Characterization to Clinical Practice. PLoS ONE.

[14] Lopez-Herrera G., Tampella G., Pan-Hammarström Q., Herholz P., Trujillo-Vargas C.M., Phadwal K., Simon A.K., Moutschen M., Etzioni A., Mory A. (2012). Deleterious mutations in LRBA are associated with a syndrome of immune deficiency and autoimmunity. Am. J. Hum. Genet..

[15] Bezzerri V., Lentini L., Api M., Busilacchi E.M., Cavalieri V., Pomilio A., Diomede F., Pegoraro A., Cesaro S., Poloni A. (2022). Novel Translational Read-through–Inducing Drugs as a Therapeutic Option for Shwachman-Diamond Syndrome. Biomedicines.

[16] Chu D., Wei L. (2019). Nonsynonymous, synonymous and nonsense mutations in human cancer-related genes undergo stronger purifying selections than expectation. BMC Cancer.

[17] Campofelice A., Lentini L., Di Leonardo A., Melfi R., Tutone M., Pace A., Pibiri I. (2019). Strategies against Nonsense: Oxadiazoles as Translational Readthrough-Inducing Drugs (TRIDs). Int. J. Mol. Sci..

[18] Hodgson S. (2008). Mechanisms of inherited cancer susceptibility. J. Zhejiang Univ. Sci. B.

[19] Garg S., Nagaria T.S., Clarke B., Freedman O., Khan Z., Schwock J., Bernardini M.Q., Oza A.M., Han K., Smith A.C. (2019). Molecular characterization of gastric-type endocervical adenocarcinoma using next-generation sequencing. Mod. Pathol..

[20] Abreu R.B.V., Gomes T.T., Nepomuceno T.C., Li X., Fuchshuber-Moraes M., De Gregoriis G., Suarez-Kurtz G., Monteiro A.N.A., Carvalho M.A. (2022). Functional Restoration of BRCA1 Nonsense Mutations by Aminoglycoside-Induced Readthrough. Front. Pharmacol..

[21] Burke J.F., Mogg A.E. (1985). Suppression of a nonsense mutation in mammalian cells in vivo by the aminoglycoside anthiotics G–418 and paromomycin. Nucleic Acids Res..

[22] Leubitz A., Frydman-Marom A., Sharpe N., van Duzer J., Campbell K.C., Vanhoutte F. (2019). Safety, Tolerability, and Pharmacokinetics of Single Ascending Doses of ELX-02, a Potential Treatment for Genetic Disorders Caused by Nonsense Mutations, in Healthy Volunteers. Clin. Pharmacol. Drug Dev..

[23] Roy B., Friesen W.J., Tomizawa Y., Leszyk J.D., Zhuo J., Johnson B., Dakka J., Trotta C.R., Xue X., Mutyam V. (2016). Ataluren stimulates ribosomal selection of near-cognate tRNAs to promote nonsense suppression. Proc. Natl. Acad. Sci. USA.

[24] Prokhorova I., Altman R.B., Djumagulov M., Shrestha J.P., Urzhumtsev A., Ferguson A., Chang C.-W.T., Yusupov M., Blanchard S.C., Yusupova G. (2017). Aminoglycoside interactions and impacts on the eukaryotic ribosome. Proc. Natl. Acad. Sci. USA.

[25] Huang S., Bhattacharya A., Ghelfi M.D., Li H., Fritsch C., Chenoweth D.M., Goldman Y.E., Cooperman B.S. (2022). Ataluren binds to multiple protein synthesis apparatus sites and competitively inhibits release factor-dependent termination. Nat. Commun..

[26] Lentini L., Melfi R., Di Leonardo A., Spinello A., Barone G., Pace A., Palumbo Piccionello A., Pibiri I. (2014). Towards a rationale for the PTC124 (Ataluren) promoted read-through of premature stop codons: A computational approach and GFP- reporter cell-based assay. Mol. Pharm..

[27] Pibiri I., Melfi R., Tutone M., Di Leonardo A., Pace A., Lentini L. (2020). Targeting Nonsense: Optimization of 1,2,4-Oxadiazole TRIDs to Rescue CFTR Expression and Functionality in Cystic Fibrosis Cell Model Systems. Int. J. Mol. Sci..

[28] Corrao F., Zizzo M.G., Tutone M., Melfi R., Fiduccia I., Carollo P.S., Di Leonardo A., Caldara G., Perriera R., Pace A. (2022). Nonsense codons suppression. An acute toxicity study of three optimized TRIDs in murine model, safety and tolerability evaluation. BioMedicine.

[29] Carollo P.S., Tutone M., Culletta G., Fiduccia I., Corrao F., Pibiri I., Di Leonardo A., Zizzo M.G., Melfi R., Pace A. (2023). Investigating the Inhibition of FTSJ1, a Tryptophan tRNA-Specific 2′-O-Methyltransferase by NV TRIDs, as a Mechanism of Readthrough in Nonsense Mutated CFTR. Int. J. Mol. Sci..

[30] Khadiullina R., Mirgayazova R., Davletshin D., Khusainova E., Chasov V., Bulatov E. (2022). Assessment of Thermal Stability of Mutant p53 Proteins via Differential Scanning Fluorimetry. Life.

[31] Crawford D.K., Alroy I., Sharpe N., Goddeeris M.M., Williams G. (2020). ELX-02 Generates Protein via Premature Stop Codon Read-Through without Inducing Native Stop Codon Read-Through Proteins. J. Pharmacol. Exp. Ther..

[32] Tanaka T., Watanabe M., Yamashita K. (2018). Potential therapeutic targets of *TP53* gene in the context of its classically canonical functions and its latest non-canonical functions in human cancer. Oncotarget.

[33] Gencel-Augusto J., Lozano G. (2020). p53 tetramerization: At the center of the dominant-negative effect of mutant p53. Genes Dev..

[34] Rizzotto D., Englmaier L., Villunger A. (2021). At a Crossroads to Cancer: How p53-Induced Cell Fate Decisions Secure Genome Integrity. Int. J. Mol. Sci..

[35] Georgakilas A.G., Martin O.A., Bonner W.M. (2017). p21: A Two-Faced Genome Guardian. Trends Mol. Med..

[36] Grover R., Candeias M.M., Fåhraeus R., Das S. (2009). p53 and little brother p53/47: Linking IRES activities with protein functions. Oncogene.

[37] Hu J., Cao J., Topatana W., Juengpanich S., Li S., Zhang B., Shen J., Cai L., Cai X., Chen M. (2021). Targeting mutant p53 for cancer therapy: Direct and indirect strategies. J. Hematol. Oncol..

[38] Li S., Li J., Shi W., Nie Z., Zhang S., Ma F., Hu J., Chen J., Li P., Xie X. (2023). Pharmaceuticals Promoting Premature Termination Codon Readthrough: Progress in Development. Biomolecules.

